# Up-to-Date Applications of Microarrays and Their Way to Commercialization

**DOI:** 10.3390/microarrays4020196

**Published:** 2015-04-23

**Authors:** Sarah Schumacher, Sandra Muekusch, Harald Seitz

**Affiliations:** Branch Bioanalytics and Bioprocesses, Fraunhofer Institute for Cell Therapy and Immunology, Am Muehlenberg 13, 14476 Potsdam, Germany; E-Mails: sarah.schumacher@izi-bb.fraunhofer.de (S.S.); sandra.muekusch@izi-bb.fraunhofer.de (S.M.)

**Keywords:** microarray, diagnostics, lateral-flow assay, drug abuse, allergies

## Abstract

This review addresses up-to-date applications of Protein Microarrays. Protein Microarrays play a significant role in basic research as well as in clinical applications and are applicable in a lot of fields, e.g., DNA, proteins and small molecules. Additionally they are on the way to enter clinics in routine diagnostics. Protein Microarrays can be powerful tools to improve healthcare. An overview of basic characteristics to mediate essential knowledge of this technique is given. To reach this goal, some challenges still have to be addressed. A few applications of Protein Microarrays in a medical context are shown. Finally, an outlook, where the potential of Protein Microarrays is depicted and speculations how the future of Protein Microarrays will look like are made.

## 1. Introduction

Microarrays play a significant role in basic research since many years and get more and more important for clinical applications and diagnostics. Different formats, detection modes and analytes are available. Microarrays are applicable in the fields of DNA, proteins, peptides and small molecules like metabolites and drugs. DNA-Microarrays were first used in research and became in the meantime a favored tool for gene expression, sequencing and detection of mutations. For this reason the actual state of this technology is quite mature. Due to diverse reasons, other applications like Protein Microarrays still struggle to achieve a routine procedure, which is essential for use as standardized service in clinics or doctors’ office.

Protein Microarrays (PMAs) gained more and more attention over the last two decades. PMAs have undergone a remarkable development since their first publications, including technological aspects like automatization and data interpretation. Since there are only a few commercially available, PMAs are still in their infancy concerning daily routine use in clinics, which use lateral flow assays and ELISA as standard procedures. Nevertheless, Protein Microarrays have the opportunity to develop into powerful tools for diagnostics, e.g., personalized medicine. This review will emphasize the history of Protein Microarrays with regard to the essentials of its development from macro to nano. Furthermore, some applications with medical background will be depicted with special focus on potential for its use on a daily routine, e.g., allergies, autoimmune diseases, and drug screening. Finally an outlook on the development of Protein Microarrays is given and challenges this technique has to overcome are outlined.

This review will not cover topics like immobilization strategies [1,2,3], standardization [4,5] and applications in cancer research [6,7] in detail, because these subjects are widely covered in literature and would go beyond the scope of this article.

## 2. History and Scientific Base

Since most PMAs use antibodies because of their specific detection of epitopes, immunoassays [8] were the first method transferred to Protein Microarrays.

**Figure 1:**
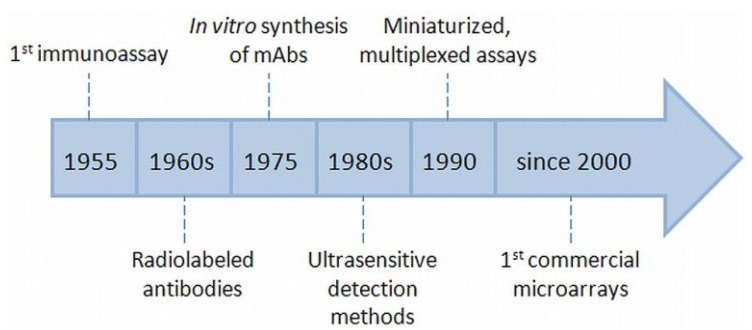
Timeline of the Protein Microarray development. Important benchmarks, which are essential for the feasibility of PMAs, are mentioned.

The very first immunoassay from Berson and Yalow [9] in 1955 marks the starting point of the development of this technology. The authors developed a method to detect Insulin with a radioimmunoassay (RIA). The technology progress was benefited when two groups developed, independently, assays with radiolabeled antibodies [10,11] in the late 1960s. An extremely important milestone was the development of *in vitro* synthesized monoclonal antibodies by Köhler and Milstein in 1975 [12]. The process was pushed further by the design of ultrasensitive detection methods in the early 1980s [13] and had another highlight in 1990 with the development of a miniaturized, multiplexed “microspot” assay by Ekins and co-workers [14,15]. This invention represents the base for the “modern” Protein Microarray [5,16]. In Figure 1, milestones that led to the development of Protein Microarrays are depicted.

The evolution of PMAs led to a huge variety of applications and experimental designs. During the last years a general classification of Protein Microarrays into three different categories became prevalent [16,17,18], (I) Analytical Microarrays; (II) Functional Microarrays; and (III) Reverse Phase Microarrays. In the following, unique characteristics of the categories are described.

**Figure 2:**
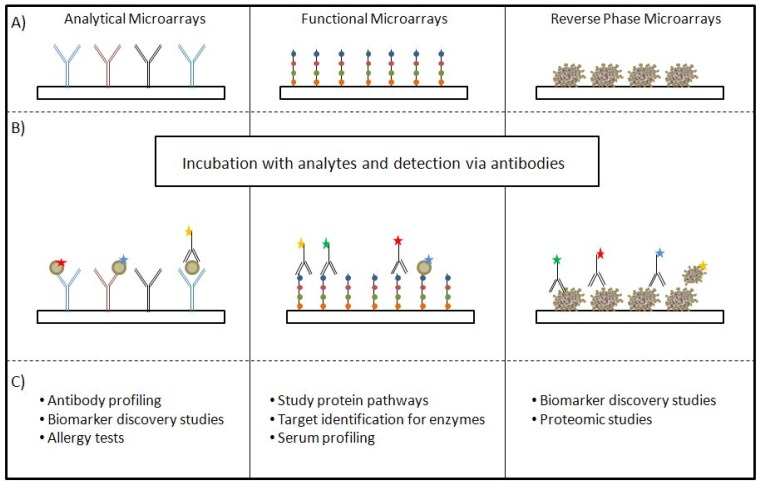
Classification of PMAs. Depicted are the different categories (**A**) and their corresponding analytes (**B**). Additionally some examples for applications are mentioned (**C**). Modified from [24,26].

Analytical Microarrays are used to measure interactions, like protein-protein interactions. Either antibodies or antigens are immobilized, incubated with a corresponding analyte and detected via an antibody, which is labeled with an enzyme or a fluorescent dye [17]. These are used for applications like antibody profiling and in biomarker discovery studies [19,20] or in a more clinical context for allergy tests or autoimmunity diagnostics [16]. The second category, Functional Microarrays, deals among others with the study of protein pathways, the identification of targets for enzymes e.g., kinases or the detection of enzymatic activity of proteins [21,22,23]. Usually proteins or protein domains are printed [17]. Reverse Phase Microarrays (RPMAs) use the most complex sample to immobilize on a solid surface. Herein cell or tissue lysate is spotted [17] for biomarker discovery or proteomic studies [16]. Due to the complex sample, RPMAs are not suited for utilization in clinical diagnostics [16]. An overview of the different categories is given in Figure 2. For more detailed information on the different classes of Protein Microarrays, have a look at *i.a.* the publication by Hultschig *et al.* [24] or Sutandy *et al.* [25].

The development of the huge varieties of applications was mainly driven by technological developments for manufacturing and processing of Protein Microarrays. Thereby, the main forces were the need to achieve better sensitivity, decrease processing times, and higher throughput [14,17]. PMAs have the advantage of a high signal-to-noise-ratio, low volume consumption, the option for multiplexing, which is an essential feature for clinical diagnostics, and in comparison to other analytical methods, PMAs are quite inexpensive [16,27]. These characteristics provide a starting point to improve existing methods or replace them in clinical routine as well as in research.

Within the last few years, the technology of Protein Microarrays underwent a remarkable development concerning the applicability and its production for basic research.

Currently, the best-studied systems are measurements of PSA and cytokines [27]. In the following, an insight into usage of Protein Microarrays in medical interrogations is given, some commercialized arrays and extraordinary applications are described and a conclusion where the development of PMAs will lead with emphasis of the application in routine diagnosis and research.

## 3. Applications

### 3.1. Application of PMAs for Small Molecules

The detection of small molecules, e.g., illicit drugs, doping agents or other metabolites, belongs to one of the most delicate methodology but gains more and more significance for stakeholders, like the food industry, sport agencies, police and law enforcement, and emergency departments of hospitals. This means there is a significant need for fast and reliable systems including point-of-need testing [28]. For doping, over 150 substances are listed as prohibited by WADA (World Anti-Doping Agency). Additionally, the number of prohibited compounds increases each year owing to production of new synthetic substances. Due to the small molecular weight and chemical properties, small molecules cannot be detected with long approved laboratory techniques like electrophoretic procedures. Therefore highly sophisticated technologies like mass spectrometry (MS) are necessary to ensure a reliable proof of these substances. Currently, the gold standard is the analysis via gas chromatography coupled with mass spectrometry (GC-MS). This technology was first introduced in the 1970s [29,30]. The advantage of this technique is the high sensitivity, but it is characterized by a low throughput, time-consuming sample preparation, and expensive equipment [31,32]. Due to complex sample preparation, MS-technology is not convenient for measurements of biological samples like whole-blood or serum. Another challenge is still the detection of more than one substance in one single assay. Although newly developed instruments tend to be easier to handle and more affordable, they are still expensive and need highly trained personnel. Other common methods are lateral flow immunoassays (LFA) or enzyme-linked-immunosorbent-assays (ELISA), which use antibodies for detection. Both are characterized by an easy protocol and quite cheap material, but often lack the required sensitivity and specificity in comparison to MS. Furthermore, for immuno-based assays, a coupling of the small molecules to a carrier molecule like BSA (bovine serum albumin) and an indirect competitive measurement is needed. Nevertheless, there is a strong progress to improve sensitivity and specificity to use immunoassay as a standard procedure because of several advantages like an easy handling, cheap reagents, and usage of more common platforms like microplates (MTP) or paper strips, which are more prevalent than MS-machines. Another important issue besides simplifying test procedures is to increase the throughput.

The issues mentioned above can be managed partly by the application of Protein Microarrays. Protein Microarrays provide a platform for multiplexed analysis, high-throughput and are in general cheaper than MS based analysis [31]. Additionally, they are potentially easier to adapt for clinical use, e.g., automatization. Furthermore, they are easier to miniaturize, including integrated microfluidics and sample preparation [28]. This can reduce reagent handling and transport, minimizes energy consumption and opens the door for small handheld devices [28,33]. Because of the lack of two different epitopes of small molecule for antibody binding, the principle of a competitive assay for screening of drug abuse and doping is used [34]. Two strategies are possible. First, for a direct competitive assay, the antibody is immobilized and incubated with a sample and a labeled conjugate. Second to perform an indirect competitive assay, the antigen, normally a recombinant compound, is immobilized and the biological sample added together with the primary antibody is incubated on the array. A secondary labeled antibody is needed for detection. In both cases relevant compounds compete for the binding sites of the specific primary antibody. A positive sample gives decreasing signals. Basically an enzymatic reaction [34,35] or a fluorescent read-out [32] can be used as detection methods.

In the following section, different immunoassays for illicit drugs and doping in a medical context are depicted and advantages and disadvantages of Protein Microarrays according to corresponding methods like ELISA and LFA are discussed.

#### Drug Abuse

Du *et al.* [31,32,36] developed a Protein Microarray for the detection of different prohibited drugs in various biological fluids. They printed up to ten different drug conjugates in triplicates onto an aldehyde modified glass slide including mouse IgG as internal control. They use a competitive approach and the described incubation steps for blocking and antibody binding lie between 30 min and one hour at 37 °C and a detection mode via fluorescence. They reported a coefficient of determination (R^2^) of 0.99 and a coefficient of variation (CV) of 16% between batches and of 13% within one batch. The presented assay was compared to the commercially available Evidence System by Randox. This system can measure 16 substances within two hours via a fluorescent detection mode with a coefficient of determination (R^2^) of 0.99. The Evidence System was validated for different applications by various laboratories and analyses of various biological matrices are possible, e.g., whole blood, serum, urine and oral fluid. Randox’ Evidence system was introduced in 2001 [31] as the first commercial multianalyte-analysis device. The detection limit of the assay developed by Randox is dependent on the drug of interest and lies between 0.2 ng/mL for Morphine and 19 ng/mL for Methadone. Furthermore, they stated storage of the PMAs for three months at room temperature. According to the mentioned results the established assay has a better performance than common ELISAs. They concluded that Protein Microarrays are able to complete existing reference methods but not to replace them. Finally, they claim that the biggest problem is cross-reactivity of the antibodies, which demand a careful screening and validation. Finding specific antibodies for small molecules is quite difficult, because the smaller they are, the more structural similarities exist between the drug and their metabolites, which make the manufacturing of highly specific antibodies complicated. Alvarez *et al.* [37] used the Evidence System to measure meprobamate in human blood samples. Meprobamate belongs to the family of carbamates, is an anxiolytic agent, and is used as a muscle relaxant analgesic with a barbiturate-like mode of action. The system is faster than a GC-MS measurement including sample preparation and analysis. But still the processing time is too slow for emergency cases. The authors of the study emphasized that in contrast to the manufacturer’s instructions, the calibrators should be analyzed in sample material instead of an aqueous buffer.

In comparison to PMAs, methods like LFA and ELISA are more mature and more spread but have significant disadvantages, e.g., more sample and reagent consumption and at the moment only a minor possibility for multiplexing. In the following some examples will be discussed.

For Lateral Flow Assays normally a nitrocellulose membrane is used. At this surface, e.g., specific gold-labeled antibodies are immobilized, which capture corresponding antigens out of the sample. The labeled antibodies flow through the membrane and get captured by associated antibodies at the test line or control line, respectively. This technique is a simple and robust rapid test, where the contact of the sample with the test strip starts all further reactions and in general is completed after approximately 10 min. Even this well-established method underlies further developments. Taranova *et al.* [38] used spotting technology in combination with LFA for drug abuse detection. They performed a competitive immunoassay on a nitrocellulose membrane on which all reagents were spotted *a priori*. The group declares a satisfying detection limit with a wide working range and a CV smaller than 10%. Like the already mentioned LFA, most of them use urine for drug detection. There are several commercialized ones like the Oratect III or the Triage DOA (Alere) [39]. All of them are easy to perform, need minimal time consumption and provide an immediate result.

Another approach is performing the whole assays in a MTP. In the case of drug screening, a competitive immunoassay has to be performed, which leads to decreasing signal intensities with increasing drug concentrations. A modified experimental setup was developed by Burks *et al.* [40]. In this approach, special dyes are immobilized in a 96-well MTP and dependent on which drug is present, the dye reacts and develops a specific color. This can be recorded with spectrophotometry and is encoded binary, which result in an unique code for each analyte. This system called DETECHIP^®^ is also available on glass slides.

Another potential application of PMAs is doping control, e.g., in various sport contests. In general screening for compounds used for doping is similar to the detection of drug abuse. That is why only a few examples are mentioned.

Beside the examples given above, the DETECHIP^®^ system can be used for steroid identification [41]. An example for performing ELISA, is the work of Tort *et al.* [42]. They performed a screening for three different steroids in human serum. They were able to achieve concentrations below the detection limit, but it is necessary to precipitate and dilute the serum. Furthermore, the same setup was tried for Microarrays, but they do not meet the required detection limits.

### 3.2. Applications of PMAs—Allergen Arrays

Type I allergies (e.g., grass, tree pollen, venoms, food) are a worldwide health problem. Twenty-five percent of the industrial world suffers from type I allergies. [43]. Type I allergies lead to a direct allergic reaction through activation of mast cells. After the first contact with allergens, the specific IgE (sIgE) level increases. The concentration of sIgE in serum/plasma correlates directly to the sensitization level of a patient. For diagnosis, the history of a patient (what he has eaten, what plants he has contacted, *etc.*) and *in vitro* diagnostics are relevant. Routinely, a low number of allergens can be tested with the prick test. prick test is a skin testing procedure, where extracts from allergens dropped onto the skin of the patient. After 5 to 60 min a visible skin reaction can be detected. Allergic symptoms are often multi sensitization effects, while using allergen extract instead of purified allergens or recombinant expressed proteins it is not possible to distinguish between cross-reactivity or species-specific co-sensitization. Many allergens have the same protein epitopes, which means that the same sIgE antibody can bind to different allergens from different sources. With the state-of-the-art allergy tests it is impossible to distinguish between them. Furthermore, skin tests can trigger overreactions of the immune system and thereby harm the patient.

To make it as comfortable as possible for the patient, different *in vitro* methods are available as diagnostic tools like ELISA, Microarrays, Lateral Flow Assays or Bead based Assays. ELISA and prick tests are the standard procedures in laboratories right now, but with these, the number of samples is limited and every allergen must be tested separately. With this kind of procedure, the sample volume is high and cross-reactivity cannot be determined. With high-throughput assays like Protein Microarrays or Bead based Systems, component resolved diagnosis and check for cross-reactivity in one assay is possible. The sample volume is much lower—up to 30 µL blood or serum—which is important especially for diagnosis of young children. Lower costs, lower sample volume and the possibility of measuring a lot more components in one experiment make high-throughput tools attractive for diagnostic and the industrial market.

Due to their shortcomings, e.g., reliability and short storage time, most high-throughput tools are only used in research and not as a diagnostic tool. Up to now, there is only one commercialized Protein Microarray diagnostic tool available, which has been used since 2008, for the identification of Type I allergies, ImmunoCAP Immuno Solid-phase Allergen Chip (ImmunoCAP ISAC) (Phadia, Thermo Scientific, Uppsala, Sweden). The development of more multiplexing assays like Bead Based or Lateral Flow Assays is in progress.

ImmunoCAP ISAC contains 112 native and recombinant allergens from 51 allergy sources, covalently immobilized on a surface. The Protein Microarray has four fields, and every field can be incubated with a different patient sample. To be sure that the there are no false positive results, serum of healthy people have been analyzed with the array [44]. The array is used in daily clinical routine and some clinical studies are done with it [45,46,47]. In these studies combined, nearly 500 clinical patients are screened with different generations of ImmunCAP ISAC. Results, in general are good analytical performance of the array, for example positive percentage of agreement between 75% to 100% and a negative percentage of agreement between 90% to 100% [46], but loss of sensitivity for a few allergic components. In all studies, the main statement is clear: the array has a good performance and gave more information about cross-reactivity and co-sensitivity in allergic persons, but cannot be used without standard tests like prick, due to the false negative results. False results are a major problem in most of the immunoassays, not only microarrays: cross-reactivity with serum components like Human-anti-mouse Antibodies (HAMAS), unspecific binding of serum components, matrix effects or sensitivity problems can occur. Even the handling, e.g., storage temperature and time of the patient probe, can lead to false results [48,49,50,51]. The analytical errors can be as high as 4% per assay [52]. Melioli *et al.* compared ImmunoCAP ISAC directly with a standard ELISA (ImmunoCAP); even though the methods are different, a high consistency could be demonstrated [53]. The same result was observed by Vigh-Conrad *et al.* in 2010, with a self-made allergen chip. Only 1% false positive results and data correlation up to 97% with standard ELISA methods has been observed [54]. Compared to the standard procedures today, there are almost no drawbacks to using Protein Arrays. It has to be discussed, how the amount of data should be handled in the future and what to do if the array data are not consistent with clinical data [55]. Even in standard clinical procedures, like double blind, placebo controlled food challenges (DBPCFC) studies, only 50% agreement is achieved with skin testing procedures [56]. There is no perfect failure free method available on the market.

In 2014 Mechanisms of the Development of ALLergy (MeDALL), a collaborative project supported by the European Union, designed a new allergen array, with 170 allergens and spotted concentrations from 50 to 200 fg. All allergens are spotted in triplicates and are purified from extract or recombinant expressed proteins [57].

In contrast to ImmunoCAP ISAC, the industrial standard, the number of allergens is extended (e.g., nuts, cashew, pistachio, cow milk, wheat allergens, olive, mite, dog, insect venom, *etc.*) and the spotted concentration is lower [57].

#### 3.2.1. MeDALL *versus* ImmunoCAP ISAC

Lupinek *et al.* compared classical immunoassays and Microarray based IgE measurements as well as the industrial standard (ImmunoCAP ISAC), and the new MeDALL chip. There is nearly no difference in sensitivity between chip based IgE measurements and quantitative measurement.

For sIgE detection, both slides were incubated with undiluted serum. The detection is achieved by adding fluorescent-labeled detection antibody. Afterwards, the slides were scanned with a confocal laser scanner. Up to concentrations of 32 ng/mL of the monoclonal antibody (mAB), a linear correlation between AB concentration (dilution series between 640 ng/mL down to 0.05 ng/mL was performed) and the signal in ISU (ISAC standardized units (for IgE-detection)) was found. With high levels of IgE, a high reproducibility was achieved (CV: 1.7%, SD: 2.26 ISU, 640 ng/mL = 132.02 ISU). The same experiment was performed on ImmunoCAP (ELISA). IgE dilutions from 0.2 to 312.5 ng/mL were tested in triplicates. A linear correlation between IgE concentration in ng/mL and UA/mL (units antigen per milliliter) up to the highest concentration could be shown. The MeDALL allergen chip shows higher CVs for low concentrations (mean CV 1 ng/mL = 14.1%, SD: 6.8%) than for higher concentrations (mean CV = 7.7%, SD = 5.5%). The same experiment was performed with the ImmunCAP ISAC; the lowest concentration was close to the background: low concentration 2.7% mean CV (SD 1.4%) and 2.3% (SD 2%). To summarize, the new MeDALL chip has a higher sensitivity and more allergen sources than the industrial standard, but in general it also has a higher number of false negative results and results must be proven with alternative techniques.

There are two other methods that should be mentioned in context of multiplexing and easy and fast allergen detection methods for clinical routine in future perspectives.

#### 3.2.2. Lateral Flow Assays (LFA)

Lateral Flow Assays (LFA) have some advantages over Protein Microarrays: they can be performed by non-specialists in the field, the results are directly visible (colorimetric detection), and the assay time is really fast [58]. Due to the colorimetric detection, no expensive documentation device is necessary. Big disadvantages right now are that only a limited number of allergens can be analyzed and the results are semi-quantitative. On the market there are a number of different LFA Assays, for example ImmunoCAP Rapid (Thermo Scientific; Waltham, MA, USA), ALFA (Dr. Fooke Laboratorien GmbH, Neuss, Germany) and Milchcheck (NanoReproAG, Marburg, Germany).

It would be great to get the fast assay time and colorimetric detection of LFAs combined with high-throughput and sensitivity of the Protein Microarrays. Therefore, in 2014 Svahn *et al.* [58,59] designed a point-of-care device, “vertical and lateral flow Allergen Microarray assay”, which is a combination of Protein Microarray and a paper-based colorimetric system. Over 1000 allergens are spotted onto nitrocellulose layers, four replicates per allergen. Due to the colorimetric detection, it is possible to detect spots with a flatbed scanner. Comparing data with these assays to the industrial standard ImmunoCAP gives a good correlation (R^2^ = 0.89, n = 31 serum samples). High sensitivity with a good reproducibility (CV < 14%) can be achieved. Due to the low costs and easy handling, combining these two methods seems to be interesting in the future for daily diagnostics.

#### 3.2.3. Bead based Allergen Test

During the last few years, bead-based assays have gained more and more attention in the field of multiplexing. Compared to the Protein Microarray technology, these assays are suspension assays. The multiplexing grade depends on the chosen assay, e.g., Luminex offer 500 different labeled beads (Luminex cooperation, Austin, TX, USA), BD Cytometric Bead Array 30 different labeled beads (BD CBA) from BD Bioscience (San Jose, CA, USA). Bead based Assays are based on the principle of flow cytometry. Generally, it is possible to bind the protein/DNA/peptide of interest to the bead surface, which are internally labeled. The beads can be mixed and measured simultaneously, which reduces the sample volume. Time of storage and multiplexing grade depends on the coupled compounds. Pomponi *et al.* [60] developed a Bead based allergen test (ABA) where allergen molecules are coupled to BD CBA functional beads. After incubation with sera, the detection of sIgE was performed with labeled secondary antibodies and a *Fluorescence-activated cell sorting* (FACS) Aria system. The given results compared to the ISAC system gave a good correlation between these systems. King and colleagues 2007 set up a seven-plex Bead based Assay for allergens and compared them to ELISA. Between these methods a good correlation R^2^ = 0.97 exist. These kinds of assays are very flexible compared to the rigid Microarrays [61]; theoretically the bead set which is used can be individualized for every patient. Other advantages are the low sample volume, which is important for pediatric diagnostics, and the short assay time. Because of this, there are projects that are trying to get Food and Drug Administration (FDA) approved Bead-based allergy tests, for example AmberGen (Watertown, MA, USA), who developed a multiplex immunoassay by adapting VeraCode™digital holographic micro-bead technology, developed by Illumina (San Diego, CA, USA) [62]. All in all it has to be considered that the mentioned platforms have their own advantages and disadvantages, but combination of the different systems can help to revolutionize the daily diagnostic in clinics.

### 3.3. Applications of PMAs—Autoimmune Diseases

Another application for Protein Microarrays is the field of autoimmune diseases like rheumatic arthritis (RA). RA is a chronic inflammatory joint disease, which causes articular cartilage and bone destruction, joint deformity and loss of mobility. To prevent those symptoms, an early diagnosis of RA is important [63]. Up to now, there are only tests that are based on the detection of a single biomarker, which are not specific only for RA or are only in a subset of patients detectable, like auto-antibodies against citrullinated peptides [64]. One example for a multiplex test system to identify auto-antibodies in human serum is Inno-Novel Line Immunoassay for the identification of antinuclear antibodies (INNO-LIA ANA UPDATE) (FUJEREBIO, Gent, Belgium). This is a strip test where 13 antigens are spotted and can be tested in one run. According to the manufacturer’s specification, the target specificity lies over 98%, combined with a high sensitivity. However, if you wish to screen for RA patients, for example you will get signals for two auto-antibodies that are not specific for RA, the patient could also have Sjögren syndrome [65]. The major problem so far is that there is no known specific biomarker profile for RA. One of the most promising therapies was the anti-TNF treatment, but only 60% of the patients responded to the medication [66]. To address the problem that there is no specific biomarker profile and a subdivision in patient groups with subtypes of RA is not possible, most of researchers are using antigen Protein Microarrays to screen for auto-antibodies, which can distinguish between different subgroups of the disease and are specific only for RA and no other autoimmune disease.

Research groups are using heterogeneous patient groups when possible, for example Hueber *et al.* [67] used patient sera from American, Swedish and Japanese people with RA; or Chandra *et al.* [48], who have 120 probes from patient with different autoimmune diseases, including RA patients, to minimize the effect of ethnic subgroups or unspecific biomarkers. Both groups are using Protein Microarrays, self-made antigen arrays or custom made ones to detect new biomarkers.

The aim of Chandra *et al.* was to develop a clinical-grade, automated system for the differential diagnosis and molecular stratification of RA. Therefore they screened serum of patients within the early phase (duration less than six months) of RA with different autoimmune diseases for auto antibodies and cytokines. The measurement of candidate antigens were performed on a chip from Roche diagnostics (Rotkreuz, Switzerland), immunological multi- parametric *chip* technique (IMPACT). IMPACT is based on a polystyrene chip, which is coated with a streptavidin layer. The candidate biotinylated antigens-peptide, proteins or antibodies are spotted in rows in duplicates onto the surface. For proceeding, the chip was incubated with 40 µL patient serum (1:10 dilution) and the binding events can be prolonged with dioxigenylated secondary antibody. The secondary antibody was then detected by a fluorescent conjugated anti-dioxigenlyated antibody. The current system can measure 100 single determinations in one run [68]. They looked for the intra-assay reproducibility by performing 21 replicate measurements of each of nine markers in on run, which gave a CV range from 1.5% to 9%, and the inter-assay reproducibility lies between 1.1% and 14.9%. The results were comparable to ELISA results. In the end, they designed an automated multiplex biomarker assay, which can distinguish between patients with RA and healthy people or individuals with inflammatory arthritides. The use of three newly identified biomarkers yielded a sensitivity of 84.3% and a specificity of 93.8% for RA [64].

Another group that is looking for a specific marker profile for RA patients with a duration less than one year is Charpin *et al.* [63]. In this study they screened the sera from patients with different duration times with ProtoArray from Invitrogen (Carlsbad, CA, USA). ProtoArray is a Protein Microarray with a nitrocellulose layer where 8268 human proteins with a GST-Tag are spotted. The proteins are purified under native conditions. For proceeding, the surface was blocked with 1% BSA and incubated with the serum. After incubation, the slides has to be washed and incubated with a detection antibody IgG Alexa Fluor 647. Data is acquired with GenePix Pro software and data evaluation is done using ProtoArray Prospector 2.0 (Invitrogen). With this method, they identified three proteins WIBG, GABARALP2 und ZNF706 to be a specific marker for early stages of RA.

These are only two examples for screening methods with Protein Microarrays, both using a combination of multiplexing techniques and Protein Microarrays to detect auto-antibodies in early stages of RA. The usage of different surface chemistry and test systems makes it difficult to compare the data from different research groups. Protein Microarrays, all in all, gave a good opportunity to screen for specific biomarker profiles in different diseases. To set up a test system for daily diagnostics in clinics there first has to be a proven biomarker profile specific for the disease; so for autoimmune diseases, a high-throughput device is still far away from daily routine use in clinics.

### 3.4. Issues of Clinical Trials

For this review a few number of clinical trials were used and compared. As has been known for a long time, researchers in many published studies failed to mention important facts like study design, sample number, and inclusion criteria. Most researchers do not validate the found antigen in a double-blind study, or do a platform comparison like Song *et al.* does [69].

Due to the bad quality of clinical trials since 2003 there are two Standards available that should improve the quality of clinical studies: Standards for reporting of diagnostic accuracy (STARD) and quality assessment of diagnostic accuracy studies (QUADAS) [70,71]. QUADAS consists of 28 quality items concerning the methodological quality of a study, while STARD has a list of 21 items concerning the completeness of reporting potential sources of bias and generalization [70,71,72]. Wilczynksi *et al.* compared journals that use the STARD guidelines with those who did not, as well as a comparison between studies before and after STARD. All in all, there was no difference between before and after STARD in clinical papers [73]. The major issue is that only a few journals are using STARD and QUADAS as guidelines for authors [70]. Although these quality guidelines exist, most studies and journals are not aware of them and the quality of clinical studies is still in a bad condition.

## 4. Conclusions

To summarize the sections of applications, the following can be said: the quality of the assay rises and falls with the quality of the applied antibody.

Each platform has its own advantages and its own drawbacks (Table 1):

- ELISA is a well-established method in a widespread format, but mostly lacks the opportunity for multiplexing and needs a high reagent volume.
- LFA is an easy-to-perform and fast method, but is often characterized by a high detection limit and an unpredictable performance.
- Protein Microarrays have the advantage of low sample consumption, the possibility of multiplexing and are normally performed on conventional glass slides. But in most cases they are expensive in the production, especially because of the technical equipment needed for dispensing and handling of very low volumes.
- The Bead Based System is the most flexible tool of the presented applications. They are applicable in personalized medicine and have a longer storage time than PMAs.

**Table 1:** Characteristics of applications.

Application	Sample Volume	Processing Time	Sensitivity	Multiplexing Grade	Field Applicability	References
**LFA**	low	<1 h	low	low	yes	[58,59]
**ELISA**	high	<5 h	middle	low	no	[31,42]
**Beads**	middle	<3 h	middle	middle	no	[60,61]
**PMAs**	low	<5 h	high	high	no	[16,27,31]

Another important fact is the influence of the utilized reagents and solutions. Most publications stated that there is a significant difference whether the method was tested with buffer or a biological sample. Therefore possible matrix interferences with the experiment have to be considered. One common point is that the antibodies have to be chosen carefully.

Especially for screening for small molecules, highly specific antibodies are needed. The differences between individual compounds are very small, which means careful screening for antibodies is required.

In comparison to that, Allergen Arrays are the most advanced concerning commercialization. Protein Microarrays are already on the market and are used in daily clinical routine. They are helpful for personalized allergen profiling, but Allergen Arrays are still very expensive. Nevertheless they can be very helpful for the treatment of children because Allergen Arrays require a lower sample volume than standard methods.

Protein Microarrays for autoimmune diseases still have their issues concerning establishment as a routine tool in clinics. More optimization has to be done and that is why they are at the moment far away from commercial use.

The biggest issue for performing immunoassays, independent of the used platform, is the already mentioned cross-reactivity. This has to be checked in detail for each antibody and even for each new batch.

## 5. Outlook

Of course there are several factors that have to be taken into account for performing Protein Microarrays, like printing and assay conditions and the later statistical analysis of the attained data, but we want to close this review on how much is possible with Protein Microarrays and to illustrate further opportunities, which are delivered by the technology of Protein Microarrays. The review by Berrade *et al.* [2] provides an excellent overview of the latest development in production of Protein Microarrays.

Microarrays already show that they have significant advantages compared to standard procedures. Very low volume of the precious sample during the assay procedure is needed. If it is possible to reach the same reproducibility of standard procedures and to reduce the production, costs Protein Microarrays are a promising tool for a number of applications.

Protein Microarrays offer a wide variety of applications, make high-throughput analysis possible and deliver a huge data amount. They are already used as routine tool in auto‑antibody-profiling, cancer research and signal pathway imaging.

We assume that the application of Protein Microarrays on glass slides will be reserved for use in research because of expensive machines for production and read-out. When the technology is transferred to platforms like MTP or LFA, it can be possible to use the combination in daily routine, e.g., clinics and doctors’ office. By using widespread platforms, it is possible to benefit from existing infrastructure and to improve diagnostics by a better sensitivity and lower sample consumption. We expect that the highest potential of Protein Microarrays lies, beside existing expertise, e.g., cancer research, in the fields of biomarker research and drug discovery. Finally, it is very important to combine the expertise of basic research and application specialists to exploit the complete potential of Protein Microarrays. It might be that as a consequence new methods will emerge and fields like personalized medicine, systems biology or biomedicine could benefit from this combination. These theses are supported by surveys of Frost and Sullivan [74,75]. They stated that Protein Microarrays in the field of personalized medicine have a high potential for growth and technical developments, especially for the pharmaceutical and biotechnology industry. It is supposed that PMAs will increase the knowledge of complex diseases and sensitize towards preventive healthcare. They predict a rapid growth till 2020 in the market sector of immune related diseases and improving medical records by optimizing efficiencies of electronic systems.
